# Deep Blue Light Amplification from a Novel Triphenylamine Functionalized Fluorene Thin Film

**DOI:** 10.3390/molecules25010079

**Published:** 2019-12-24

**Authors:** Tersilla Virgili, Marco Anni, Maria Luisa De Giorgi, Rocio Borrego Varillas, Benedetta M. Squeo, Mariacecilia Pasini

**Affiliations:** 1 IFN-CNR, c/o Dipartimento di Fisica, Politecnico di Milano, P.zza Leonardo Da Vinci 32, 20132 Milano, Italy; 2 Dipartimento di Matematica e Fisica “Ennio De Giorgi”, Università del Salento, via per Arnesano, 73100 Lecce, Italy; 3 SCITEC-CNR, Via Corti 12, 20133 Milano, Italy; squeo@ismac.cnr.it

**Keywords:** lasing, organic molecule, ultrafast spectroscopy, Amplified Spontaneous Emission, optical gain, fluorene

## Abstract

The development of high performance optically pumped organic lasers operating in the deep blue still remains a big challenge. In this paper, we have investigated the photophysics and the optical gain characteristics of a novel fluorene oligomer functionalized by four triphenylamine (TPA) groups. By ultrafast spectroscopy we found a large gain spectral region from 420 to 500 nm with a maximum gain cross-section of 1.5 × 10^−16^ cm^2^ which makes this molecule a good candidate for photonic applications. Amplified Spontaneous Emission measurements (ASE) under 150 fs and 3 ns pump pulses have revealed a narrow emission at 450 nm with a threshold of 5.5 μJcm^−2^ and 21 μJcm^−2^ respectively. Our results evidence that this new fluorene molecule is an interesting material for photonic applications, indeed the inclusion of TPA as a lateral substituent leads to a high gain and consequently to a low threshold blue organic ASE.

## 1. Introduction

Due to their good optical properties and low-cost large-area processing, organic π-conjugated semiconductors have gained much interest in different kinds of applications such as organic field-effect transistors (OFETs) [1,2,3] light-emitting diodes (OLEDs) [4,5], solar cells (OPVs) [6,7] and semiconductor lasers [8,9,10,11]. Although much progress has been made in optoelectronic devices such as OLEDs, conjugated molecules, to date, neither oligomers nor polymers have found applications in real laser devices, either optically or electrically pumped. Particularly, the great challenge of developing novel molecules with highly luminescent efficiencies, efficient optical gain, high photostability and low lasing thresholds in order to achieve electrically pumped organic lasers remains open [12]. To close this gap between the actual state of the art and the performances required for real applications, research on the development of novel materials for organic lasers is still ongoing. In particular, one of the open issues in this frame is the realization of organic molecules with good gain properties in the blue-near UV spectral range that would be strategic blue-laser-light therapy against antibiotic-resistant bacteria [13], as well as for excitation source in spectroscopic experiments.

Among the large class of semiconducting organic conjugated materials, fluorene derivatives are promising compounds for organic lasers because of their efficient blue emission and lasing characteristics with low threshold [14,15,16,17,18]. However, most of the fluorene-based materials suffer from chemical instability due to easy oxidation with formation of fluorenone moieties responsible for the appearance of a low energy green emission band [19]. Another issue that needs to be addressed is their aggregation during time responsible for morphology evolution from amorphous to different forms of aggregation including, crystalline phases (i.e., α and αù^’^ phase) and non crystalline phases (nematic, and β phase) [20,21,22,23].

Indeed, the long alkyl chains, added as lateral substituent to improve solubility and film forming ability, can rearrange leading to aggregation which can be responsible for excimer formation or exciton quenching and increased energy transfer (ET) toward the green fluorenone (or keto) defects [24,25]. All these factors must be eliminated in order to obtain a material with good stability, low threshold and large gain suitable for deep blue light amplification.

Triphenylamine (TPA) moiety has been proposed as a versatile building block able to suppress both keto defects and aggregation during time [26,27]. Hole-transporting molecules such as TPA derivatives are reported to act as antioxidants for conjugated materials and to inhibit the oxidation of fluorene derivatives, hence considerably reducing the concentration of keto defects [18,28]. The bulky and propeller-shaped TPA structure incorporated into the polymer backbone tends to be packing-disruptive, most of the TPA substituted materials are amorphous, with high thermal stability, good film-forming ability, and good solubility [29,30,31]. Moreover, the propeller like structure of TPA is responsible for a reduced interchain ET to residual fluorenone defects process normally more efficient than the intrachain one [32]. In this way, the color stability of the material is increased. The introduction of TPA in OLED active materials has been extensively studied both as a unit in the conjugated backbone [32,33] and as a lateral substituent [34], leading in all cases to considerable improvements in the material [35,36,37,38] performances. Regarding materials for light amplification, the effect of TPA moiety has been instead evaluated only as terminal unit of the main backbone [39] and not yet as lateral substituent.

**Figure 1:**
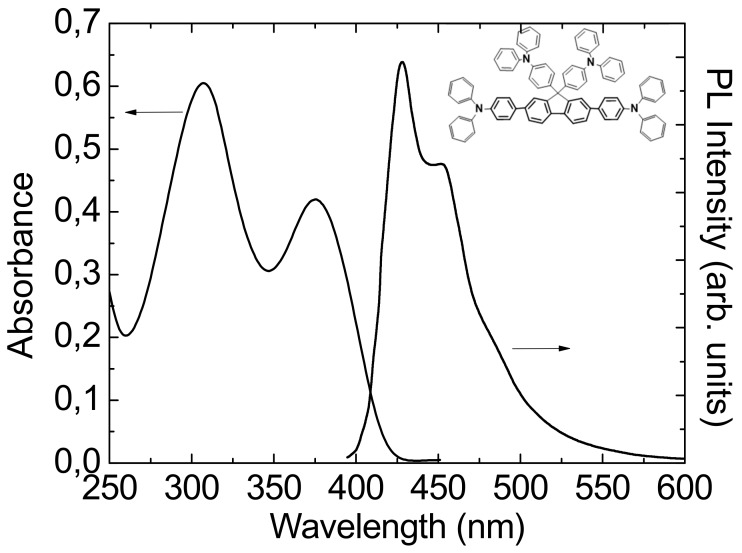
Absorbance and Photoluminescence spectra of the thin film. The chemical structure of the new fluorene-oligomer with four TPA substituents F4TPA is shown in the inset.

In the present work we have evaluated the TPA impact as a lateral substituent and we have synthesized a new fluorene-oligomer with four TPA substituents, F4TPA, reported in Figure 1. We have explored the photophysics of a F4TPA thin film by ultrafast spectroscopy and its gain properties by studying the Amplified Spontaneous Emission (ASE) both under impulsive and quasi steady state (QSS) pumping. We have demonstrated that the molecule has a broad Stimulated Emission (SE) spectral band between 400 and 500 nm with a maximum cross-section at 450 nm of 1.5 × 10^−16^ cm^2^. The thin film presents ASE emission both under impulsive and QSS pumping with a low threshold of 5.5 μJcm^−2^ and 21 μJcm^−2^, respectively. The ASE threshold under QSS pumping is about 2 times smaller than the one found in molecules having the same number of phenyl units in the conjugated backbone but different lateral substituents, evidencing the potentiality of the TPA lateral substituents for the design of blue high gain materials.

## 2. Results and Discussion

A spin-coated film of the F4TPA from toluene solution (15 mg/mL) on a fused silica substrate was prepared with a thickness of 120 nm. The absorption spectrum of the film (see Figure 1) shows a first peak at 377 nm and a higher energetic one at 307 nm, while the two main peaks of the photoluminescence spectrum are at 425 nm and at 450 nm with a shoulder at about 480 nm. The PL spectrum results to be stable in air with no appearance of the typical green large emission mainly due to the keto defect. This result is consistent with previous reports [18,22] evidencing that the introduction of TPA group as substituent on 9-position of a fluorene unit reduces the number of fluorenone defects as a result of its relevant antioxidant action. In fact, it is generally accepted that keto-defects are generated in PFs by the oxidation of residual monoalkylfluorenes [37] already present in the polymer (monoalkylated fluorene residues present as impurities incorporated into the polymer during polymerization), or generated during operation by thermal-, photo-, or electro-oxidative degradation processes catalyzed by the presence of residual metal impurities [38] of some PFs. Anyway, the origin of green emission remains the subject of long standing debate and it has been attributed also to morphological degradations that induce material changes resulting in low-energy excimer formation. This hypothesis has been discussed in a recent work [20] indicating that the dominant mechanisms behind the appearance of the green band in PFO are different forms of aggregation, including H-aggregation and possibly charge transfer or excimer formation. In this context it is clear that the bulky TPA substituents do not allow for this aggregation, in contrast to smaller and more flexible aliphatic substituents, e.g., alkyl chains.

In order to evaluate the presence of high gain and of interchain interactions responsible for charge formation, ultrafast transient absorption spectroscopy has been performed on the film.

**Figure 2:**
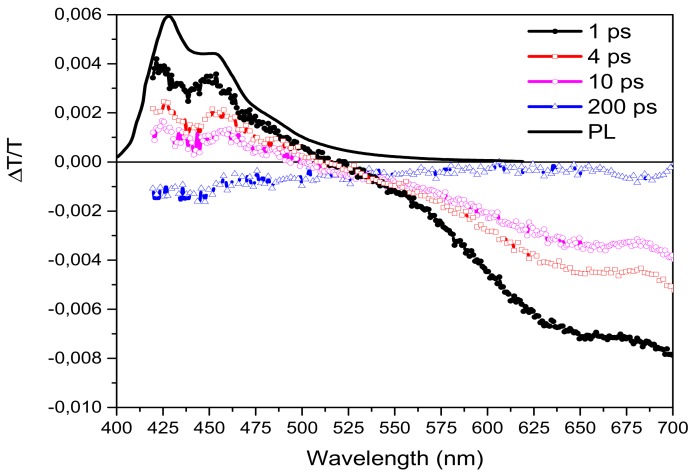
Transient differential transmission spectra at different probe delays. Photoluminescence spectrum is shown for comparison.

In Figure 2 we report the chirp-free differential transmission (ΔT/T) spectra of F4TPA molecule for different time delays between the pump and the probe after excitation at 400 nm with a fluence of 0.5 mJ/cm^2^. The measured signal is:

(1) $$\frac{\Delta T\left(\lambda ,\tau \right)}{T}\;=\;\frac{T_{pump}-T_{no\;pump}}{T_{no\;pump}}$$

where T_pump_ and T_nopump_ are the probe transmission intensities after and before the pump excitation at a given λ and τ (probe delay), respectively. A positive ΔT/T signal is an evidence of bleaching (BL) of the ground state absorption or of stimulated emission (SE) from excited states, while a negative signal indicates the presence of a photoinduced absorption (PIA) process. The spectrum at 1 ps time delay (black dots in Figure 2) shows the change in the probe transmission spectrum soon after the pump excitation. In the spectrum at this delay, a positive signal between 420 nm and 520 nm and one photoinduced absorption (PIA) band over 520 nm, with a peak around 650 nm are present. The positive signal perfectly overlaps with the photoluminescence spectrum (see the black solid line in Figure 2), so it can be easily ascribed to the stimulated emission signal from the excitons singlets population (S1→S0) [40]. At longer probe delays, the expected decrease of the signal is evident all over the spectrum, with a particular change of sign in the blue spectral region where the initial positive ΔT/T signal becomes negative after 200 ps (blue line in Figure 2).

**Figure 3:**
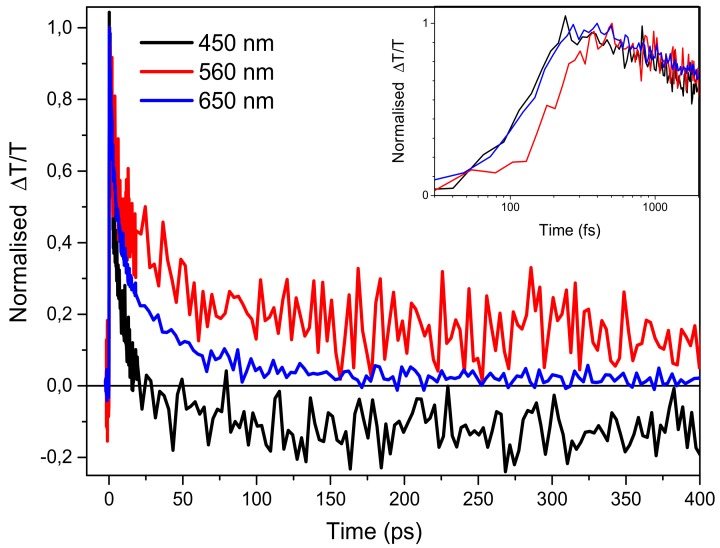
Temporal decays at different wavelengths. In the inset zoom of the first picosecond probe delay.

In order to understand the origin of the instantaneous negative signal (peak at 650nm) and of the delayed negative one present in the high energy spectral region, we have compared the time decays at 560 nm and at 650 nm (see Figure 3) with the one at 450 nm already attributed to the singlets stimulated emission by the comparison with the PL spectrum. The three dynamics are all different, but, while the ones at 450 nm and at 650 nm are instantaneously created (in our time resolution of around 150 fs), the one at 560 nm is delayed by ~350 fs (see inset of Figure 3). The different temporal decays at 560 nm and 650 nm indicate that we can spectrally distinguish between two PIA bands: one partially overlapping with the SE emission (450–580nm) visible at long probe delays and another one peaked at 650 nm and extended until 700 nm instantaneously created. We attribute the latter to photoinduced absorption from the first singlet state S_1_ to a higher one S_n_ (S_1_→S_n_), while the one at 560 nm to the generation of charged states after 350 fs from the pump pulse excitation (see inset in Figure 3). We exclude the presence of triplet states due to the fast generation time not possible in an intersystem crossing mechanism especially in molecules without the presence of heavy metals. The signal at 450 nm has an initial decay similar to the one at 650 nm but, after around 20 ps, the signal becomes negative indicating that at this probe delay the negative contribution in the signal due to charges becomes predominant on the fast positive one.

**Figure 4:**
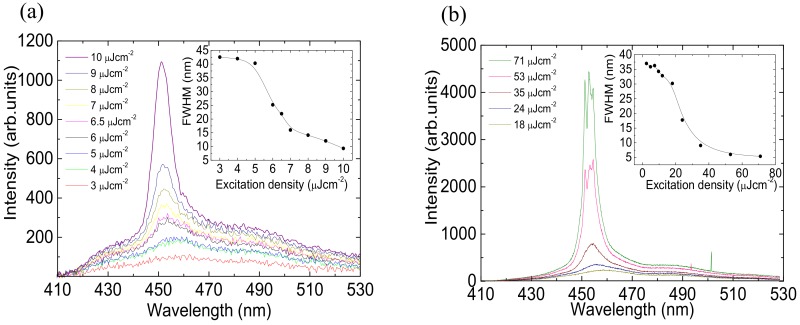
Amplified Spontaneous Emission (ASE) measurements: (**a**) Photoluminescence spectra at different fs pump excitation density (**b**) Photoluminescence spectra at different ns pump excitation density. The insets show the change of the spectra linewidth as a function of the excitation density.

We note that, due to the low contribution of the photoinduced charge absorption in the SE spectral region, this molecule can be considered a good candidate for photonic applications. We, then, have measured the initial cross-section of the stimulated emission transition (σ) at 450 nm, using the following formula [40]:

(2) $$\frac{\Delta T}{T}\left(450\;nm,\;150\;fs\right)\;=\;\sigma N_{ph}$$

where N_ph_ represents the number of photons absorbed per unit of area, calculated as E_p_/(SE_λ_) (where E_p_ is the pump pulse energy absorbed, E_λ_ is the energy of each pump photon and S the pumped spot area). The cross-section is calculated to be 1.5 × 10^−16^ cm^2^ with N_ph_ = 33.75 × 10^12^ cm*^−^*^2^. This value is comparable to the one of high gain organic molecules [41]. In order to further investigate the optical gain properties of F4TPA we also performed PL measurements with a rectangular excitation spot size as a function of the excitation density both under ultrafast (150 fs) and QSS (3 ns) pumping. The photoluminescence spectra as a function of the excitation energy density under ultrafast pumping are reported in Figure 4a (inset). At low excitation density, the spectra are consistent with the F4PTA spontaneous emission and, as the excitation density increases above 5 μJ/cm^2^, a clear narrow band appears at about 450 nm, progressively dominating the spectra. This feature is typical of ASE, with an estimated threshold of about 5.5 μJ/cm^2^, corresponding to the excitation density at which the ASE narrow peak starts to appear and its linewidth starts to decrease (see Figure 4a inset). The ASE sharp peak at 450 nm, corresponds to the 0–1 vibronic emission peak (Figure 1), as often observed in organic molecules and consistent with the four-vibronic-level gain process, allowing low threshold optical gain. No degradation has been observed during the measurements. The low ASE threshold value under ultrafast pumping further stimulated our interest toward the investigation of the ASE properties in QSS pumping conditions, potentially compatible with simple optical pump sources, like UV laser diodes [42,43], and thus particularly relevant in order to evaluate the applicative potentiality of the molecule in realistic pumping conditions of a cheap organic laser.

The excitation density dependence of the PL spectra under QSS pumping, reported in Figure 4b, allows us to observe the spontaneous emission spectrum al low excitation density and the appearance of a clear ASE band centered at 450 nm with a threshold of 21 μJcm^−2^. A fine structure with narrow peaks is superimposed in this case to the ASE band, likely due to scattering assisted random lasing [44]. Also, in this case, the sample does not show degradation during the measurements.

**Figure 5:**
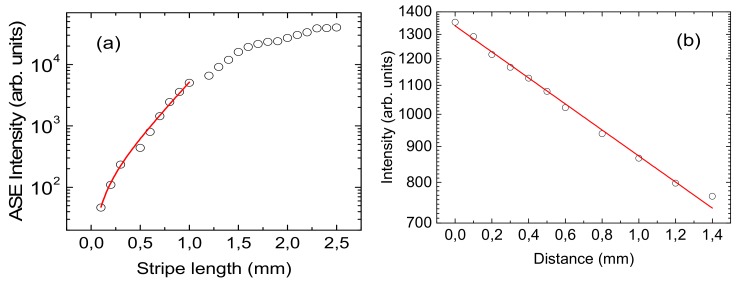
(**a**) Experimental ASE intensity peak measured at different stripe lengths (open circles), and best fit curve with equation 3 (red line) (**b**) ASE intensity peak measured changing the distance between the excitation stripe and the sample edge (open circles), and best fit curve with Equation (4).

The net gain value at 450 nm has been determined by the Variable Stripe Length method, by measuring the spectral evolution as a function of the stripe length, at a fixed excitation density of 350 μJ/cm^2^, under QSS pumping, reported in Figure 5a The output intensity of the excited stripe follows the relation [45]:

(3) $$I(\lambda ,L)=\frac{I_{0}(\lambda )}{g(\lambda )}\left[e^{\left(g(\lambda )L\right)}-1\right]$$

where I(λ, L) is the PL intensity at a given wavelength λ and stripe length L, I_0_(λ) is the spontaneous emission intensity per unit of stripe length and g(λ) is the net gain value at wavelength λ. A gain value of 40±2 cm^−1^ has been obtained from the best fit of the experimental emission intensity dependence on the stripe length at the ASE peak wavelength. We have to point out that g(λ) is the net gain of the material, given by a positive contribution (ASE) and a negative contribution due to all the loss mechanisms of the waveguide [46]. For this reason, we have also determined the waveguide propagation losses, by measuring the PL intensity dependence at the ASE peak on the stripe distance from the sample edge, at fixed stripe length (1 mm) and excitation density (70 μJ/cm^2^). The data show a clear exponential decrease (see Figure 5b, to note the *y*-axis is in the log scale) as the stripe distance from the edge increases, consistent with the theoretical dependence:

(4) $$I\left(\lambda ,d\right)=I_{0}\left(\lambda \right)e^{-\alpha d}$$

A losses value of 4.3 ± 0.1 cm^−1^ has been determined from the best fit of the experimental data. This result allows us to estimate the absolute g (450 nm) value of around 45 cm^−1^ [32,46,47,48,49].

**Figure 6:**
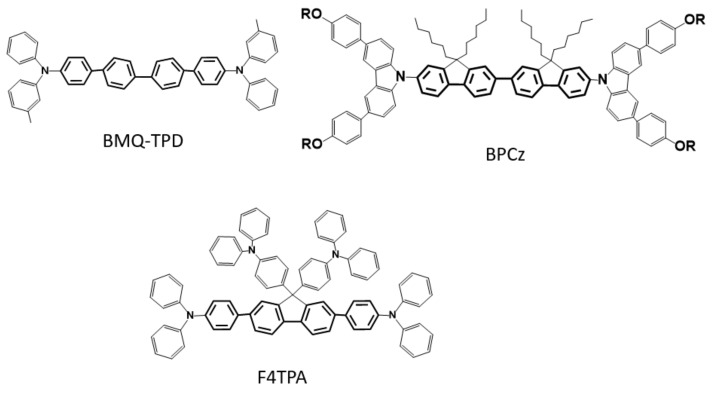
The molecular structures of the BMQ-TPD, a diphenyl oligomer with two TPA derivatives, and BPCz, a bisfluorene with terminal biphenylcarbazole units, molecules. The structure of our molecule is shown for comparison.

In order to correctly compare our results with those obtained from other similar molecules it is important to observe that the ASE threshold strongly depends on the pump pulse time length and also on the phenyl rings number [11,47] in the main backbone. We thus compare our results with the ones obtained in two molecules with the same number of phenyl rings but different lateral substituent (the chemical structures are reported in Figure 6). In particular the BMQ-TPD a diphenyl oligomer with two TPA derivatives [39] does not have lateral substituents while the BPCz, a bisfluorene with terminal biphenylcarbazole units [11,48] have four hexyl alkyl chains, moreover these molecules have terminal groups containing nitrogen and phenyl rings as in F4TPA.

The ASE thresholds were reported under nanosecond pumping, from net molecular film in the case of BPCz and from polystyrene blend film for the BMQ-TPD. The values are calculated to be around 50 μJ/cm^2^, thus more than 2 times larger than the one we have found for the F4PTA. This comparison strengthens the idea that the inclusion of TPA as a lateral substituent of a fluorene leads to a better gain and a lower threshold and it is more efficient than the alkyl chains which do not prevent deleterious aggregation or oxidation of the molecule.

## 3. Materials and Methods

### 3.1. Molecule Synthesis

All reagents and solvents are used as received or purified using standard procedures. Tetrakis(triphenylphosphine)palladium (Pd(Ph_3_P)_4_) and Triphenylamine-4-boronic acid (compound **1**) available from Aldrich are used as received. Flash chromatography purifications were carried out using silica gel (200–300 mesh ASTM), Suzuki reactions with conventional heating are carried out under nitrogen atmosphere and 9,9-bis(4-diphenylaminophenyl)-2,7-dibromofluorene (compound **2**) is prepared according to literature [50]. To a schlenk tube 267 mg (0.92 mmol) of compound **1**, 300 mg (0.37 mmol) of compound **2**, and 8.5 mg (2% mol) of (PPh_3_)_4_Pd are introduced. Then 5 mL of anhydrous toluene, 3.4 mL of a degassed solution of 2 M K_2_CO_3_ and a catalytic amount of triethylbenzylammoniumchloride (TEBA) are added under nitrogen to this mixture. The reaction solution is heated at 90 °C under stirring for 24 h. The crude product is filtered on Celite, washed with water, and dried. Flash chromatography of the residue (silica gel, hexane/dichloromethane 8/2) afforded the title compound (390 mg, 93% yield). Anal. Calcd for C86H66N4: C, 89.39; H, 5.76; N, 4.85%. Found: C, 89.12; H, 5.1; N, 5.76%. ^1^H NMR (600 MHz, CDCl_3_): *δ* = 7.79 (d, 2H), 7.62 (d, 2H), 7.57 (d,2H), 7.45 (d, 4H), 7.28 (t, 8H), 7.20(t, 8H), 7.14 (m, 16H), 7.04 (m, 12H), 6.97 (t, 4H), 6.91(d, 4H).

### 3.2. Steady State Absorption and Photoluminescence Spectra

Absorption spectra have been collected by a Varian Cary 500 dual beam spectrophotometer. Steady-state emission are obtained using an FLS 980 (Edinburg Instrument Ltd., Livingston, UK) and a Nanolog (Horiba Scientific, Piscataway, NJ, USA) spectrofluorimeter. The excitation wavelength for the emission spectra was 390 nm.

### 3.3. Ultrafast Spectroscopy

The ultrafast spectroscopy setup is fed by a 150-fs, 2-kHz repetition rate Ti:sapphire system (Libra, Coherent, Santa Clara, USA) with a central wavelength of 800 nm. Transient Absorption measurements were performed by pumping at 400 nm with the second harmonic of the laser output, generated with a 1-mm, Type I β-barium borate crystal. The pump energy was adjusted at 75 nJ, providing a fluence of 0.5 mJ/cm^2^ with a pump spot area of 0.16 mm^2^. The probe pulses, with a spectrum spanning from 450 to 750 nm, were obtained by white-light generation in a 3-mm thick sapphire crystal. The measurements were performed in transmission and the probe spectrum was detected using a SP2150 Acton, Princeton Instruments spectrometer. The pump beam was modulated by a mechanical chopper at 1 kHz frequency and the differential transmission (ΔT/T) spectrum of the probe was measured as a function of probe wavelength and pump-probe delay. The polarization between pump and probe beams was set at magic angle (54.7°).

### 3.4. ASE Measurements with Femtosecond Excitation

ASE was obtained by pumping the film at 400 nm with the second harmonic of the Ti:sapphire system (150 fs pulse duration) with a rectangular stripe spot excitation with length of 1.05 mm and width of 0.140 mm (1.5 × 10^−3^ cm^2^). The emitted spectrum was collected at the edge of the film with the help of a spectrograph (Princeton Instruments SP2150, Acton, MA, USA, 300 gr/nm) coupled with a CCD camera (Princeton Instruments pixis 256). The spectral resolution was 0.5 nm. All the measurements were performed in air.

### 3.5. ASE Measurements with Nanosecond Excitation

The ASE and optical gain measurements on the films under nanosecond pumping were performed by using an LTB MNL 100 nitrogen laser, delivering 3 ns pulses at 337 nm, with a peak energy up to 155 μJ as excitation source. The pump laser has been focused by a cylindrical lens on a rectangular stripe with length up to 4 mm and width of 80 µm. The laser excitation density has been varied by a variable neutral filter. The samples emission was collected from the sample edge, after waveguiding along the pumped stripe, spectrally dispersed by an Acton 750 spectrometer, and detected by an Andor Peltier cooled CCD. The spectral resolution was 0.5 nm. All the measurements were performed in vacuum (10^−2^ mbar).

## 4. Conclusions

In conclusion, we have investigated the photophysics and the optical gain characteristics of a novel fluorene molecule functionalized by four triphenylamine groups. We have found a broad gain region from 420 nm to 500 nm. Measurements of ASE under 150 fs and 3 ns pump pulses allowed us to observe a clear ASE band at 450 nm, with a low threshold of 5.5 μJcm^−2^ and 21 μJcm^−2^, respectively. The calculated gain cross-section of 1.5 × 10^−16^ cm^2^ and an absolute gain value at 450 nm of ~45 cm^−1^ make this molecule a good candidate for deep blue photonic applications. By comparing our molecule with others similar reported in literature we can conclude that TPA moiety has a chemical structure suitable for the development of high gain materials. Furthermore, the typical green emission of fluorenone derivatives is not observed, thanks to a twofold action of the TPA able to prevent fluorene oxidation, thanks to TPA recognized relevant antioxidant action, and at the same time to prevent aggregation, thanks to its bulky and propeller-shaped structure.
